# 

**DOI:** 10.1038/sj.bjc.6600381

**Published:** 2002-01-02


0.33③M (+/-0.13③M) in a cell-free assay[1]. RHPS4 is a novel pentacyclic acridine (3,11-difluoro-6,8,13-trimethyl-8H-quino[4,3,2-kl]acridinium methosulphate) synthesised at Nottingham.
The aims of the study were to investigate the pharmaceutical and biological properties of RHPS4 to assess whether it was a suitable drug for further development and to validate telomerase inhibitory effects by RHPS4 at the pre-clinical, in vitro stage. RHPS4 has been shown to possess convenient pharmaceutical properties: high aqueous solubility (>5mg/ml) was determined by UV absorbance and its stability under tissue culture conditions confirmed by HPLC assay. Fluorescence activated cell sorting has shown rapid cell uptake of the drug in the breast and lung cancer cell lines MCF-7 and A549 respectively. Saturation of drug uptake is approached after approximately six hours (MCF-7 cells) or two hours (A549 cells) at 37°C; uptake is much slower at 4°C. Naturally fluorescent, RHPS4 can be visualised in the nuclei of these cells within 30 minutes by fluorescence confocal microscopy.
Concentrations causing low acute cytotoxicity (□1③M as determined by four-day MTT assays) have been used in the study of the biological effects of the drug to avoid toxic effects not attributable to telomerase inhibition. In longer-term studies of cell viability, concentrations □1③M cause the proportion of dividing cells to decrease with time of treatment. Further, RHPS4 induces a senescent phenotype in a proportion of the cell population beginning after 8 days in culture. The proportion of senescent cells increases with time of treatment consistent with the expected effects of telomerase inhibition. In contrast, apoptosis appears not to be a predominant feature of the drug's mechanism of action, as determined by cell cycle analysis (absence of a pre-$G_1$ peak). In conclusion, RHPS4 represents a promising small molecule inhibitor of this important anticancer target.

[1] Gowan SM et al Mol Pharmacol 2001 60: 981-988

**P264**

**EFFECTS OF FLAVOPIRIDOL AND 5-FLOROURACIL ON MCF-7 HUMAN BREAST CANCER CELLS**

Gebril N*, Al-Sakkaf KA, Brown BL and Dobson PRM.
Institute for Cancer Studies and Institute of Endocrinology, Division of Genomic Medicine, Sheffield University, Medical School, Sheffield, UK

Flavopiridol (FP) is a novel cyclin-dependent kinase (CDKs) inhibitor. It is currently under phase II clinical trial. Several studies have shown that FP inhibits the growth of many human tumour cells and also causes an early specific decline in cyclin D1 in MCF-7 human breast cancer cells (1). In this work, our aim was to evaluate the effects of FP in combination with currently used drug 5-Fluorouracil (5-FU) on MCF-7 cell line. We have treated MCF-7 breast cancer cells with FP and 5-FU individually and in combination using different concentrations.
The apoptotic effects of FP and 5-FU were measured using different methods including, *In situ* nick translation assay and Annexin V assay. In addition, the apoptotic nuclei were clearly visualised on fluorescent microscope using 4,6-diamidino-2-phenylindole (DAPI) stain. Results of *In situ* nick translation and Annexin V assays were obtained by flow cytometry. Cells were counted using a coulter counter and the effects of both drugs monitored every half-hour by time lapse microscopy.
The maximum growth inhibition achieved after 48 hours when 5-FU (0.01μM) was given two hours after FP (0.1μM) was 61%, where as FP (0.1μM) and 5-FU (0.01μM) individually caused 50% and 39% growth inhibition respectively. However, the effect of this combination was not much different (63%) when 5-FU was given 24 hours after FP.
After treatment of the cells with the same drug combination, 45% of the cells undergo apoptosis. Interestingly, only 9% of the cells were apoptotic after treatment of the cells with a much higher concentration of FP alone (0.5μM), more cells undergo late apoptosis as shown by Annexin V assay. Similarly, 5-FU alone at concentration of (1μM) leads to apoptosis in only 11% of the cells.
In summary, these results showed that the effects of FP and 5-FU on MCF-7 breast cancer cells using these combinations did not give any sigh of synergism, unlike the lung cancer cell line A549 (2). In addition, the killing mechanisms in both of the drugs might not be completely apoptotic.

1-Carlson *et al*, 1999. Cancer Research 59: 4634.
2-Bible & Kaufmann, 1997. Cancer Research 57: 3375.

**P265**

**A PHASE I/II STUDY OF SU5416, A TYROSINE KINASE INHIBITOR IN PATIENTS WITH VON HIPPEL-LINDAU SYNDROME.**

S.Madhusudan*[1], E. Cattell[2], Pat Price[3],M. Tsolomas[4], Niall Moore[5], Susan Huson[6], Chris Adams[7], Peggy Frith[8], Paul Scigalla[9] , Adrian L.Harris[10].Cancer ResearchUK Medical Oncology Unit[1,2,10],Departments of Radiology[5] Genetics [6] Neurosurgery[7] and Ophthalmology[8], Oxford Radcliffe Hospitals, Oxford, Christie Hospital, Manchester[3], Birmigham &Midland Eye Clinic, Birmingham[4], U.K, SUGEN Inc, San Francisco, USA[9].

**Introduction**: Von Hippel-Lindau (VHL) syndrome is a rare autosomal dominant familial cancer syndrome caused by germline mutations in the VHL tumour suppressor gene causing transcriptional activation of vascular endothelial growth factor(VEGF)gene and aberrant angiogenesis, leading to early onset retinal angiomas, cerebellar and spinal haemangioblastomas, renal cysts, and risks of renal cancer and other solid tumours. Retinal blindness, severe neurological morbidity, and the need for bilateral nephrectomy and transplantation are the difficult issues in the management of these patients. Patients with VHL are candidates for a chronic anti -angiogenic treatment strategy. Feasibility of such a therapy and the long-term toxicity of anti-angiogenic drugs currently under evaluation in solid tumours is largely unknown and VHL patients could provide valuable information for future cancer trials. We initiated a phase I/II trial of chronic therapy with SU 5416, a tyrosine kinase inhibitor and a potent inhibitor of VEGF mediated Flk-1 signaling, in VHL patients. **Objectives and methods**: The primary objectives of the study were to assess safety, tolerability, response rates, serial changes in disease activity and surrogate markers of angiogenesis inhibition following chronic therapy with SU5416. Patients with proven VHL syndrome were eligible for the study if they had symptomatic VHL not amenable to standard therapy, or multiple renal lesions likely to lead to transplantation or progressive disease, with good performance status, adequate renal, hepatic and bone marrow profiles. Patients received SU5416 at a dose of 145 $mg/m^2$ given intravenously (initial cohort of 2 patients received once weekly and the rest twice weekly). Each cycle consisted of 4 weeks of treatment. Toxicity and disease evaluation was done 4 and 12 weekly respectively. Biological marker analysis was done pre-treatment, weeks 4, 8 and 12. **Results**: A total of 6 patients were entered into the study (3M and 3F) with a median age of 34.5 years (range 26-42). Prior to entry into the study, 6 patients had neurosurgery and 2 patients retinal laser therapy. All patients had retinal angiomas,and/or CNS haemangioblastomas and/or renal cysts. A total of 51 cycles of therapy was administered with an average of 8.5 cycles per patient (range 1.5-22.4 cycles). Three patients achieved stabilization of disease whilst on therapy lasting for 20.6, 16.3 and 5.5 months respectively. There was <50% reduction of a lumbar spinal lesion. There was complete regression of retinal lesion in one patient. The treatment was not as well tolerated as in cancer, with 1 patient discontinuing for pruritus and another having 50% dose reduction for pruritus. **Conclusions**: SU5416 was well tolerated. Chronic anti- antiangiogenic therapy is feasible and safe in this small group of patients. Regression of retinal lesion was seen in one patient though no PR/CR were seen. Future studies will be important with chronic oral therapy initiated early, since the late stages here had scarring and secondary problems that may not be blocked by VEGF antagonism .

**P266**

**A MULTICENTRE PHASE II STUDY OF SU5416, A TYROSINE KINASE INHIBITOR, IN LOCALLY ADVANCED OR METASTATIC MELANOMA**

S. Madhusudan* [1], J.Baselga[2], P.F.Conte[3], H.M. Pinedo[4], J. Braybrookes[5] Adrian.L.Harris[6]. Cancer Research UK Medical Oncology Unit, Churchill Hospital, Oxford, U.K[1, 5, 6], Hospital General Universitari Vall d'Hebron, Barcelona, Spain[2], Ospedale Santa Chiara, U.O.Oncologie Medica, Pisa, Italy[3], Vrije Universiteit Medical Center, Amsterdam, The Netherlands[4].

 © 2002 Cancer Research UK

**Introduction:** Preclinical and human pathology data support the role of vascular endothelial growth factor(VEGF) in the growth and metastasis of melanoma and this correlates with a poor prognosis. Suppression of VEGF causes tumour regression in animal models. SU5416 is a tyrosine kinase inhibitor and a potent inhibitor of VEGF mediated Flk-1 signalling. Pharmacokinetic and toxicity data is available from several phase I studies. An international phase II study was initiated to investigate the role of SU5416 in locally advanced or metastatic melanoma. **Objectives and methods:** The primary objectives of the study were to assess tumour response, duration of response and progression free survival. Analysis of possible biological markers of angiogenesis inhibition and assessment of toxicity were the secondary objectives. Inclusion criteria included histologically proven progressive stage III or IV melanoma not amenable to standard therapy, ECOG performance status 0-2, adequate renal, hepaticand bone marrow profiles. Eligible patients received SU5416 at a dose of 145 $mg/m^2$ given as an intravenous infusion on days 1 and 4 of every week. Each cycle consisted of 4 weeks of treatment with responding patients continuing treatment for upto 1 year. Disease evaluation was performed after four and eight weeks of treatment and two monthly thereafter. Toxicity assessment was done at two weekly intervals. Blood samples for assessment of biological markers were collected on days 1, 4, 8, 22 and 4 weekly from then on. **Results:** A total of twenty patients were entered into the study( 10 males and 10 females) with a median age of 53.5 years (range 23-71). ECOG performance status of 0 (four patients) and 1(sixteen patients). Prior to entry into the study, eighteen patients had received surgery, eighteen patients had systemic therapy [ immunotherapy (INF- alpha, IL-2 and others) and/or chemotherapy ( DTIC and others) and/or tamoxifen] and five patients had radiotherapy. A total of 42 cycles of therapy was administered with an average of 2.05 cycles per patient. Sixteen patients were evaluable for disease response. Six patients (37.5%) had stable disease whilst on therapy lasting less than three months and all others had progressive disease. Median time to progression was 41 days. Treatment was well tolerated with asthenia, headache, nausea, vomiting and anorexia as common side effects. Analysis of surrogate markers of angiogenesis inhibition is pending at this time.**Conclusions:** SU5416 was well tolerated and side effects were as expected from previous studies. Single agent SU5416 seems ineffective in this group of heavily pre treated patients with a high tumour burden. Future studies in combination with immunotherapy or chemotherapy or blockers of other angiogenic pathways are likely to be more promising.

### P267

### PILOT STUDY: IMMUNISATION WITH MELANOMA ASSOCIATED PEPTIDES AND GM-CSF IN HLA-A2 PATIENTS WITH METASTATIC MELANOMA.

R.Molife*[1] M.Muthana[2], D. Wilkinson[2], S.Hedley[2], S.Browne[1], R. Brookes[2], A. Murray[2], I. Rennie[2] and P.Lorigan[1]

[1]Dept of Oncology, Weston Park Hospital, Sheffield, S10 2SJ, UK, [2]Institute of Cancer Studies, Royal Hallamshire Hospital, Sheffield, S10 2JF.

The prognosis for patients with metastatic melanoma is poor. Palliative chemotherapy does not significantly improve survival and new approaches to therapy are urgently required. Evidence suggests that melanoma can stimulate specific humoral (resulting in antibody production) and cellular (via stimulation of cytotoxic T cells [CTLs] by antigen on the surface of antigen presenting cells [APC]) immune responses. Peptide epitopes derived from melanoma-associated antigens are targets for CTLs in the context of HLA-A2. Immunisation of patients with melanoma using these peptide epitopes may elicit an adequate immune response resulting in lysis of tumour cells. Immune adjuvants such as granulocyte-macrophage colony stimulating factor (GM-CSF) may augment this immune response.

AIM: The primary objectives were to i) determine immunological response as determined by delayed tissue hypersensitivity (DTH) reactions at the site of injections and induction of CTLs as measured by a chromium release assay, and ii) to define the safety and toxicity profile of vaccination. Clinical response was documented as a secondary endpoint.

METHODS: After obtaining informed consent, eligible patients were vaccinated with 100micrograms of each of 3 synthetic peptides – tyrosinase, gp-100 and melan-A (intradermal) with GM-CSF adjuvant (intramuscular) weekly for 4 weeks followed by a 4 week break (1 cycle) for up to 3 cycles. DTH reactions were assessed at 48h after the first vaccination of each cycle. Venous samples for CTL assays were obtained at intervals pre- and post-each vaccination cycle. Tumour responses were assessed clinically and radiologically after each 8-week cycle. Approval for the study was obtained from the local ethics committee.

RESULTS: An initial 9 patients with a median age of 54 were recruited. Four patients had soft tissue and/or nodal metastases, 2 had lung metastases and 3 had liver metastases. Two patients demonstrated DTH reactions to gp100 and melan-A respectively with associated CTL reactivity against target cells pulsed with the relevant peptide. Both of these patients (one with soft tissue disease, the other with lung metastases) had stable disease after 3 and 2 cycles of treatment respectively. Vaccination was associated with mild transient pain at the sites of injection; GM-CSF injection resulted in grade 1 local reaction in 4 patients.

CONCLUSION: Vaccination using melanoma-associated peptides with GM-CSF adjuvant is well tolerated and capable of eliciting immune responses that may be associated with clinical responses in selected patients with metastatic melanoma.

### P268

### THE BIOCHEMICAL AND ANTINEOPLASTIC EFFECTS OF AMIDOX, A NEW INHIBITOR OF RIBONUCLEOTIDE REDUCTASE, ON HL-60 HUMAN PROMYELOCYTIC LEUKEMIA CELLS

Zsuzsanna Horvath*, Wolfgang Bauer, Thomas Höchtl, Doris Karl, Thomas Szekeres, Monika Fritzer-Szekeres

Clin. Institute for Med. & Chem. Laboratorydiagnostics, University of Vienna, Medical School, General Hospital of Vienna, Waehringer Guertel 18-20, A-1090 Vienna, AUSTRIA

Amidox (3,4-dihydroxybenzamidoxime), a new polyhydroxy-substituted benzoic acid derivative, is a potent inhibitor of the enzyme ribonucleotide reductase, which catalyses the de novo synthesis of DNA. It is considered to be an excellent target for anti cancer chemotherapy, as its activity is significantly increased in malignant tumor cells. In our study we investigated the biochemical and antineoplastic effects of amidox. First, we incubated HL-60 human promyelocytic leukemia cells with various concentrations of amidox for 48 and 96 hours. Amidox inhibited the growth of HL-60 cells in a growth inhibition assay with an IC50 of 30 µM. In a soft agar colony forming assay, amidox yielded a 50% inhibition of colony formation at 20 µM. In addition we investigated the effects of amidox treatment on the formation of deoxynucleosidetriphosphates, which are the precursors of DNA. Amidox (75 and 100 µM for 24 hours) could significantly decrease dCTP, dGTP and dTTP pools and caused an increase of the intracellular dATP concentration. We also tested the combination effects of amidox with Ara-C, a widely used compound for the treatment of leukemia. This combination yielded additive cytotoxic effects both in growth inhibition and soft agar colony formation assays. This effect was due to the increased formation of Ara-CTP, the active metabolite of Ara-C after preincubation with amidox. This was shown by measuring the intacellular Ara-CTP concentrations using HPLC. Preincubation of HL-60 cells with 75 and 100 µM amidox for 24 hours caused an increase in the intracellular Ara-CTP concentrations by 576% and 1143%, respectively. We conclude that amidox is capable to inhibit the growth and colony formation of HL-60 cells at concentrations which can be achieved in vivo.The compound decreases dNTP pools and caused a concomitant increase of Ara-CTP. Therefore this compound might be further investigated in in vivo studies for the treatment of leukemia.

### P269

### STRUCTURALLY DISTINCT SMALL MOLECULE BAK BH3 PEPTIDE MIMETICS EXHIBIT COMPARABLE BCL-2 SENSITIVE, PRO-APOPTOTIC EFFICACY

Dean A. Fennell*, Arben Pallaska, Margherita Corbo, Finbarr E. Cotter

Department of Experimental Haematology, Barts and The Royal London, Queen Mary's School of Medicine and Dentistry, London, E1 2AD

Programmed cell death (Apoptosis) is a common biochemical process underlying the pharmacodynamics of cytotoxic chemotherapy. Intrinsic neoplastic cellular resistance to apoptosis remains a major obstacle limiting efficacy. Multidomain death agonist proteins of the Bcl-2 family Bax and

© 2002 Cancer Research UK

Bak, are prerequisite components of the core apoptotic machinery activated by chemotherapy, and target mitochondria. Bcl-2 and its multidomain homologue Bcl-$X_L$, widely expressed in several malignancies, heterodimerize to death agonists Bax and Bak, inhibiting their proapoptotic action on mitochondria. We have investigated the relative proapoptotic activity of three recently discovered, structurally unrelated, small molecule Bcl-2 antagonists, ethyl 2-amino-6-bromo-4-(1-cyano-2-ethoxy-2-oxyethyl)-4H-chromene-3-carboxylate (HA14-1), 2' methoxy-antimycin A3 (2MAMA3), and 5-benzylidine-a-isopropyl-4-oxo-2-thioxo-3-thiozolidinacetic acid (BH3I-1). These Bak peptide mimetics (BBPMs) bind Bcl-2 with micromolar affinity, preventing Bax-Bak heterodimerization, and induce apoptosis. BBPM induced dissipation of FCCP sensitive mitochondrial membrane potential was measured at single cell resolution in RAMOS Burkitt lymphoma cells using an amphipathic fluorophore $DiOC_6(3)$ and propidium iodide. Cumulative frequency distributions (CDFs) of tolerance measured at 24, 48, and 72 hours, were modelled by non-linear regression using the maximum likelihood method. A corresponding rank order of median tolerance was determined with maximum likeihood error estimates as HA14-1 > 2MAMA3 > BH3i-1. This rank order exhibited strong positive correlation (Pearson's r > 0.95) with published Bak peptide-Bcl-2/Bcl-XL binding affinities. Apoptosis measured by changes in morphology, Z-DEVD.fmk inhibitable outer plasma membrane phospholipid unpacking (using MC540), and caspase 3 activation, accompanied mitochondrial depolarization. Ramos cells transfected with full length Bcl-2 cDNA (Ramos/Bcl-2), exhibited significant tolerance to VP16, reactive oxygen species ($H_20_2$, and tert- butylhydroperoxide), and VP16 compared to vector only transfected control cells. Furthermore, Bcl-2 transfection shifted the CDF of tolerance to mitochondrial depolarization to the right, for all the BPPMs studied. Potency ratios of median tolerance for Bcl-2 transfected versus vector only transfected lymphoma cells, were of low magnitude; HA14-1 (2.5), 2MAMA3 (1.6), BH3I-1 (2). In summary, this study has quantitatively compared for the first time, the proapoptotic activity and Bcl-2 sensitivity of first generation small molecule Bcl-2/Bcl-$X_L$ antagonists. Studies to investigate interactions with chemotherapy, and pro-apoptotic pharmcodynamics, are in progress. Such pharmacophores could provide novel leads for therapeutically reversing of Bcl-2 mediated chemoresistance in cancer.

**P270**

**EVALUATION OF PHARMACODYNAMIC ENDPOINTS OF ANTITUMOUR BENZOTHIAZOLES**

Chee-Onn Leong[1]*, Margaret Gaskell[2], Peter B. Farmer[2], Michael C. Bibby[3], Patricia A. Cooper[3], John A. Double[3], Valentina Trapani[1], Tracey D. Bradshaw[1] and Malcolm F. G. Stevens[1]. [1]Cancer Research Laboratories, School of Pharmaceutical Sciences, University of Nottingham, Nottingham NG7 2RD, U.K. [2]Cancer Biomarkers and Prevention Group, The Biocentre, University Road, Leicester, LE1 7RH. [3]Cancer Research Unit, School of Life Sciences, University of Bradford, Bradford, BD7 1D, UK.

The novel antitumour agent, 2-(4-amino-3-methylphenyl)-5-fluorobenzothiazole (5F 203) elicits potent and selective anticancer activity *in vitro* and *in vivo*. The growth of MCF-7 and IGROV-1 cells is inhibited ($GI_{50}$ < 10 nM) whereas MDA-MB-435 cells are inherently resistant ($GI_{50}$ > 100 μM). When MCF-7 and MDA-MB-435 xenografts are transplanted s.c. in opposite flanks of the same mouse, the growth of MCF-7 tumours only is significantly inhibited by 5F 203. The dihydrochloride salt of lysyl amide prodrug of 5F 203 (Phortress) has been formed through conjugation with the exocyclic primary amine function. *In vivo*, Phortress significantly inhibits the growth of IGROV-1 and MCF-7 tumours. Its unique mechanism of action includes bioreversion to parent amine in the presence of tumour cells, ligand binding to cytosolic aryl hydrocarbon receptor (AhR) in sensitive cells, receptor translocation, formation of protein complexes on the CYP1A1 promoter, induction of CYP1A1 mRNA, activity and protein expression with NADPH-dependent covalent binding to CYP1A1. Subsequent formation of reactive electrophilic species lead to generation of lethal Phortress-derived DNA adducts. Putative pharmacodynamic (PD) endpoints undergoing preclinical evaluation include examination of AhR translocation, CYP1A1 protein expression, PET studies and formation of DNA adducts in tumour cells and tissue following treatment with Phortress. *In vitro*, 1μM Phortress generated 1 major and a number of minor adducts in MCF-7 and IGROV-1 cells. These adducts were chromatographically identical to those generated in DNA of MCF-7 cells following exposure to 5F 203. *In vivo*, Phortress-derived DNA adducts were detected in MCF-7 and IGROV-1 xenografts. Striking selective formation of DNA adducts in MCF-7 xenografts tissue only, distinguished sensitive (MCF-7) tumours from insensitive (MDA-MB-435) tumours transplanted in opposite flanks of the same animal. Comet assays, alkaline elution and fluorescence analyses of DNA unwinding (FADU) have confirmed massive DNA damage in sensitive (e.g. MCF-7) cells following treatment with 5F 203. Our studies are consistent with the hypothesis that this class of agent ultimately exerts its biological activity through covalent binding with DNA. The techniques studied offer potential for evaluation of a PD endpoint which may be predictive of tumour sensitivity of Phortress. Phase I clinical trials of Phortress are scheduled to begin in autumn 2002, under the auspices of CRUK.

**P271**

**THE INFLUENCE OF SOMATOSTATIN ANALOG ON SERUM CHROMOGRANIN A AND PROGRESSION OF DISEASE AMONG PATIENTS WITH NEUROENDOCRINE CARCINOMA, CARCINOID TYPE: A DESCRIPTIVE STUDY**

*Gardner, N., [1, 4] Foulis, PR., [2,3,4] FAN, Z., [4] Kvols, L., [1, 3]
[1]H. Lee Moffitt Cancer and Research Institute, [2]James Haley Veterans Administration Hospital [3]University of South Florida College of Medicine, [4]University of South Florida College of Public Health Department of Epidemiology, Tampa Florida, USA

**Background:** Malignant carcinoid tumors are rare and often associated with a specific syndrome, more than 70% are functional. Hormones and proteins that are released and associated with the syndrome are co-stored and co-released with Chromogranin A (CgA). CgA has been shown to be a sensitive and specific marker for carcinoid tumors, regardless of the tumors' functional status. The mechanism of release from the tumors may be partly exocytotic, and a positive biological gradient appears to reflect an increasing tumor load and therefore may be an independent marker of prognosis. Carcinoid tumors express a variety of receptors for somatostatin (SSTR), of which there are five known subtypes. The somatostatin analog, octreotide, binds with a high affinity to the SSTR2, SSTR5 and to a lesser degree SSTR3. SSTR2 is predominantly expressed and known to be related to a clinical benefit from octreotide therapy for the relief of the syndrome symptoms. SSTR3 and SSTR5 have known apoptotic and cytostatic roles. Octreotide may interact with the receptors to directly mediate the trigger that produces an inhibition of cell division and, indirectly, by the suppression of growth factors and hormones that stimulate tumor growth. **Research Question:** Primary – Is there a difference in the predictive value of chromogranin A levels for tumor progression among patients treated with octreotide compared to those not treated? Secondary – Does octreotide alter time to progression of disease? **Methods:** For the purpose of this abstract, retrospective data on a random 30% sample (n=76) patient population at H. Lee Moffitt Cancer Center were evaluated from the time of diagnosis to progression. Medical records, pathology slides, clinical data, laboratory results, and radiographic imaging were used for this descriptive study. **Results:** Changes in CgA level were evaluated using logistic regression while controlling for other known and suspected prognostic variables. The differences between the treatment groups OR=2.2 (.619, 7.979) and the Chi Square test was not significant. Although, 68% had at least a 2 fold increase, the OR was not significant for difference between the groups OR=1.806 (.512, 6.363). Using Cox proportional hazard ratio for survival difference, the duration of disease stability, while controlling for debulking surgery, hepatic artery embolization, chemotherapy, tumor grade, symptoms, and metastatic disease, between the two treatment groups was not statistically different (p = 0.2227 >0.05). **Conclusions:** There was not an overall survival difference demonstrated based on octreotide therapy; this may be due to the difference of disease severity at time of diagnosis and the impact of the secreted hormones responsible for the syndrome among the octreotide group. The major weakness of this study was the inconsistency of historical data, and sample size. A randomized clinical trial is needed to further evaluate benefit related to octreotide therapy.

© 2002 Cancer Research UK

**P272**

**A RECOMBINANT FUSION PROTEIN WITH POTENTIALLY REDUCED IMMUNOGENICITY FOR ADEPT**

A Mayer[1]*, DIR Spencer[1], B Tolner[1], D Purdy[2], RHJ Begent[1], K Chester[1]
[1]Department of Oncology, Royal Free Campus, Royal Free and University College Medical School, London, UK, [2]Department of Molecular Microbiology, Centre for Applied Microbiology Research, Porton Down, UK

Antibody-directed enzyme prodrug therapy (ADEPT) is based on the selective generation of cytotoxic drugs within tumours due to administration of an antibody-enzyme conjugate prior to the prodrug. Previous work using a chemically conjugated antibody-enzyme fusion protein (Napier *et al*, 2000) showed that immunogenicity hampers repeated administration. A new clinical trial uses MFE-CP, a recombinant fusion protein of an anti-CEA scFv antibody (MFE-23) and an enzyme (carboxypeptidase G2, CPG2). Since MFE-CP is a recombinant molecule there is potential to mutate immunogenic sites. A clinically relevant discontinuous conformational epitope on CPG2 has been identified and mutation of a single amino acid led to a variant with reduced immunogenicity (Spencer *et al*, 2002). Here we report the development of a new variant (dmMFE-CP) with a second mutation in the CPG2 discontinuous epitope and potential for greater reduction in immunoreactivity.

DmMFE-CP was expressed in *Eschichia coli*, purified by affinity and size exclusion chromatography with a final yield of 100ug/l supernatant. Purity was confirmed by SDS-PAGE and western blotting. Binding to CEA was confirmed by ELISA. Crucially dmMFE-CP retained intact catalytic activity, i.e. 210U/mg despite 2 amino acid changes. DmMFE-CP was then cloned into pPICZ B for expression in *Pichia pastoris*, the host for the currently used clinical product. A hexahistidine-tag for purification on immobilized metal affinity chromatography and an additional *XbaI* site were also inserted. *EcoRI* and *SalI* restriction enzyme digestion of dmMFE-CP was carried out prior to amplification by polymerase chain reaction and TOPO TA subcloning, which allowed blue-white screening to determine the correct insert. The insert was religated into the original vector puc119, digested with *XbaI* and *SfiI* and ligated into pPICZ B. The construct was transfected into *P. pastoris*, cultured in glycerol containing medium prior to induction with 1% methanol. Harvesting at 24 hours and a culture temperature of 25°C was found to give the highest yield of intact fusion protein, i.e. approximately 10mg/l. DmMFE-CP will now be tested for its immunogenic potential in comparison with the currently used clinical product MFE-CP.

Napier MP, Sharma SK, Springer CJ *et al*, 2000, Clin Cancer Res, 6, 765.
Spencer DIR, Robson L, Purdy D *et al*, Proteomics, *in press*

**P273**

**NOVEL LEAD ANTITUMOUR STRUCTURES FROM OXYGENATED AND HYDROXYLATED BICYCLIC HETEROCYCLES**

G Wells[1], JM Berry[1], TD Bradshaw[1], AM Burger[2], CS Matthews[1], MFG Stevens[1], AD Westwell[1], DA Vasselin[1]*, [1]Cancer Research Laboratories, School of Pharmaceutical Sciences, University of Nottingham, NG7 2RD, U.K.; [2] Tumor Biology Center, University of Freiburg, Breisacherstr. 117, D-79106, Freiburg, Germany

The synthesis of new oxygenated and hydroxylated heterocycles has been an ongoing area of involvement in our laboratories due to our interest in novel phenolic oxidation products[1]. These products include 4-heteroaryl-4-hydroxycyclohexa-2,5-dien-1-ones, otherwise known as heteroaromatic quinols, a new class of antitumour agent found to possess highly unusual patterns of *in vitro* activity. Antitumour activity is concentrated in certain colon and renal tumour cell lines at the $LC_{50}$ level (NCI sixty cell line human tumour screen). For example, in HCT116 and HT29 colon cell lines, 4-(benzothiazol-2-yl)-4-hydroxycyclohexa-2,5-dien-1-one (NSC 706704, a benzothiazole quinol) exhibits $LC_{50}$ values of 0.47 μM and 0.65 μM respectively (two day assay). *In vivo* activity against human RXF 944XL renal xenografts in nude NMRI mice has also been observed (complies with UKCCR guidelines).

Two major synthetic approaches to this new series of agents have been developed. The first approach to benzothiazole quinols involves phenolic oxidation of 2-(4-hydroxyphenyl)benzothiazoles using hypervalent iodine oxidants. A more versatile approach, however, to a range of heteroaromatic quinols involves lithiation of the appropriate heteroaromatic building block followed by addition of the resulting nucleophile (e.g. 2-lithiobenzothiazole) to protected benzoquinone ketal. Ketal deprotection then gives the required heteroaromatic quinol in one-pot in good yields. This methodology has provided efficient access to a wide range of substituted quinols, e.g. benzothiazole, benzoxazole, benzothiophene, benzofuran, thiazole, thiophene and quinoline.

We have adopted an information-intensive approach towards the elucidation of possible mechanistic targets for this series based on COMPARE analyses within the NCI database ($GI_{50}$ panel). For active compounds within this series Pearson Correlation Coefficients (PCCs) were found to be > 0.7, indicating a conserved mechanism of action. However, the activity profile of compound did not correlate well with standard clinical agents in the database (http://dtp.nci.nih.gov). Further COMPARE analysis of NSC 706704 revealed a number of compounds in the database for which the PCC was > 0.7 including a naturally occurring flavones-derived quinol[2]. Target COMPARE analysis uncovered the redo-sensitive protein thioredoxin as a potential target. This has been validated by experimental observations (low μM $IC_{50}$ values vs. thioredoxin). Based on the flavone compound correlation and our synthetic interest in isoflavonoids, we have initiated a program towards the synthesis of new isoflavone-derived quinols, the results of which will also be reported.

Supported by Cancer Research UK and the University of Nottingham

[1] Wells G, Bradshaw TD, Diana P, Seaton A, Shi DF, Westwell AD, Stevens MFG, (2000) Bioorg. Med. Chem. Lett. **10**: 513
[2] Wada H, Fujita H, Murakami T, Saiki Y, Chen CM, (1987) Chem. Pharm. Bull. **35**: 4757

**P274**

**DMU-212: A NOVEL CYP1B1 ACTIVATED ANTICANCER PRODRUG**

G.A. Potter, P.C. Butler*, K.C. Ruparelia, T. Ijaz, N.E. Wilsher, E. Wanogho, H.L. Tan, T.T.V. Hoang, L.A. Stanley, and M.D. Burke, Cancer Drug Discovery Group, School of Pharmacy, De Montfort University, Leicester LE1 9BH.

The cytochrome P450 isoform CYP1B1 has been shown to be expressed in a high proportion of human tumours[1], but not in the corresponding normal tissues, making this enzyme an attractive target for cancer therapy. We have designed the novel antimitotic prodrug DMU-212 using the pharmacophore of estradiol[2], the only known endogenous substrate for CYP1B1. DMU-212 has been shown to be essentially non-toxic to MCF-7 breast cancer cells or normal human breast cells (either primary cultures or MCF-10A) which lack CYP1B1, but has been shown to be highly cytotoxic to breast tumour cells expressing CYP1B1 (MDA-468 or TCDD induced MCF-7), with an *in vitro* therapeutic window of several thousand fold using a 96 hour drug exposure time. A very high tumour selectivity has been observed for DMU-212, and in the test systems used here we have obtained a tumour selectivity ratio of 9,000-fold. The cytotoxic effects on cells expressing CYP1B1 could be seen after a drug exposure time of only 15 minutes. Toxicity to the expressing cells could be reversed to that observed in non-expressing cells using the CYP1 family inhibitors acacetin or α-naphthoflavone, confirming the mechanism of bioactivation. These results suggest that DMU-212 is a non-toxic and tumour selective anticancer agent with therapeutic potential for the treatment of CYP1B1 expressing tumours, and tumours that are resistant to conventional chemotherapy.

[1]Murray, G.I., Taylor, M.C., McFadyen, M.C.E., McKay, J.A., Greenlee, W.F., Burke, M.D. & Melvin, W.T. (1997) *Cancer Res.*, **57,** 3026-3031.
[2]Potter, G.A., Patterson, L.H. & Burke, M.D. (2001). Aromatic hydroxylation activated (AHA) prodrugs. *US Patent 6,214,886.*

**P275**

**A NOVEL SUBCLASS OF THALIDOMIDE ANALOGUE WITH ANTI-SOLID TUMOUR ACTIVITY IN WHICH CASPASE DEPENDENT APOPTOSIS IS ASSOCIATED WITH ALTERED EXPRESSION OF BCL-2 FAMILY PROTEINS**

J Blake Marriott[1*], Ian A. Clarke[1], Anna Czajka[1], Keith Dredge[1], Kay Childs[1], Hon-Wah Man[2], Peter Schafer[2], Sowmya Govinda[2], George W. Muller[2], David Stirling[2] and Angus G. Dalgleish[1].

© 2002 Cancer Research UK

[1]Division of Oncology, St George's Hospital Medical School, Cranmer Terrace, London, SW17 0RE, UK. [2]Celgene Corporation, 7 Powder Horn Drive, Warren, NJ, USA.

Thalidomide is clinically useful in a number of cancers. Anti-tumour activity may be related to a number of known properties, including anti-tumour necrosis factor (TNF)-α, T cell co-stimulatory and anti-angiogenic activities. However, it may also involve direct anti-tumour effects. A series of second generation thalidomide analogues have been separated into two distinct groups of compounds each with enhanced therapeutic potential, i.e.; SelCIDs™, which are phosphodiesterase (PDE) type IV inhibitors, and IMiDs™ which have unknown mechanism(s) of action. We report here our efforts to determine direct anti-tumour effects of thalidomide and compounds from both groups. We found that one of the SelCID analogues (SelCID-3) was consistently effective at inhibiting tumour growth and viability in a variety of solid tumour lines. The anti-tumour activity was independent of known PDE4 inhibitory activity and did not involve cAMP elevation. Growth arrest was preceded by the early induction of G2/M cell cycle arrest, which led to caspase 3 mediated apoptosis. Apoptosis was associated with increased expression of pro-apoptotic proteins and decreased expression of anti-apoptotic bcl-2. Furthermore, extensive apoptosis *in vivo* was detected during SelCID-3 mediated inhibition of tumour growth in a murine xenotransplantation cancer model. Our results suggest that this analogue represents a novel class of compound with distinct anti-cancer potential.

**P276**

**THE *IN VIVO* DISTRIBUTION OF A CEA DIRECTED ANTIBODY CONJUGATED TO THYMIDINE PHOSPHORYLASE,**

C.L. Graham*, H.D. Thomas, G.A. Taylor, D.R. Newell, R. Melton#, A.V. Boddy.
*NICR, Framlington Place, Newcastle University, Newcastle upon Tyne, NE2 4HH. #Enact Pharma Plc, Building 115, Porton Down Science Park, Salisbury, SP4 0JQ.

Raltitrexed (RTX) is a classical thymidylate synthase (TS) inhibitor used to treat colorectal cancer. Inhibition of TS by RTX leads to depletion of dTTP. Tumours may overcome TS inhibition through thymidine (TdR) salvage by conversion of endogenous TdR to thymidylate using thymidine kinase. To block TdR salvage extracellular TdR can be depleted by thymidine phosphorylase (TP). The carcinoembryonic antigen (CEA) is expressed at low levels on adult colon epithelial cells but overexpressed by the majority of colon carcinomas. To achieve tumour-specific TdR depletion TP was conjugated to the anti-CEA antibody A5B7, and the distribution of the A5B7-TP conjugate studied in tumour bearing mice.

Colorectal carcinoma cells (LoVo, which express high levels of CEA) were subcutaneously injected into the right flank of nude mice (1 x $10^7$cells/animal). When the tumours had grown to 1cm$^3$ the antibody-enzyme conjugate was administered intravenously at a dose of 1mg/animal (equivalent to a TP activity of 20-25 mmoles of thymine formed per minute). Twelve, 24, 36, 48 and 72h after injection of the conjugate plasma, tumour, liver, kidney, colon, spleen, brain and lung were collected. TP activity was determined by HPLC and the protein content by a BCA protein assay. There were no changes in TP activity in any of the tissues other than the plasma and tumour, at any of the time points studied as compared with controls. TP activity expressed as the rate of thymine formation per μg protein in plasma and tumour are given in the table

| Hour post admin | 0 | 12 | 24 | 36 | 48 | 72 |
|---|---|---|---|---|---|---|
| Plasma | 0,0 | 33.3 | 7.4 | 7.6 | 1.2, 1.8 | 0.4 |
| Tumour | 2.3, 2.0 | 6.6 | 8.4 | 5.5 | 3.3, 2.3 | 2.8 |
| Significance p< | 0.06, 0.01 | 0.008 | ns | 0.02 | ns, ns | 0.001 |

These results show that the conjugate does accumulate in the tumour but not in any other tissues. However, high levels in the plasma may result in systemic toxicity when RTX is administered. Further experiments will investigate methods to optimise conjugate delivery to the tumour and to reduce levels in plasma in order to define a time-window for the administration of RTX.

This work is funded by a Cancer Research UK, William Ross Studentship.

**P277**

**PEPTIDE NANOTECHNOLOGY: APPLICATIONS IN TARGET VALIDATION AND DRUG DISCOVERY**

Rob Woodman and Paul Ko Ferrigno*
MRC Cancer Cell Unit, Hutchison/MRC Research Center, Hills Rd, Cambridge CB22XZ

Our goal is first to streamline the validation of cancer drug targets and then to apply the information gained to the identification of small molecule drugs. To these ends, we are constructing molecular toolkits that we use to study cellular proteins in their natural environment and *in vitro*.

We screen a library of $10^9$ constrained peptides expressed in eukaryotic cells for those that bind to the protein under investigation. Typically, a screen of 3 million peptides yields 30-100 hits that bind to the target protein with affinities between 1 and 300 nM. Peptide sequences are used for *in silico* screening of databases for homologous sequences that may provide insights into protein partners. Constrained peptides are expressed in cells with the goal of disrupting protein-protein interactions. Any phenotype produced in this way provides clues about functions of the target protein. We also use the peptide sequence to replace the substrate recognition domain of enzymes, creating artificial enzymes whose sole substrate is the protein of interest. We can thereby direct post-translational modifications such as ubiquitination of the target protein. Using such a designer enzyme, we can drive the proteolytic degradation of a target protein in human cells. Our next goal is to make protein knockouts in human cells. We also make functional protein knockouts by using the cell's own sorting machinery to remove the target protein from its compartment, preventing interactions with its normal partners. In biochemical studies, GST-fusions to constrained peptides can be used to purify the target protein from human cells, yielding information about the protein's in vivo interactions. These GST fusions can also be used to probe Western blots of whole cell lysates. GFP fusions to constrained peptides may also be used as molecular tracers to monitor the intracellular dynamics of the target protein in living cells. Finally, in the first case where we have a constrained peptide binding to a clinically important surface of a target protein, we have initiated a screen for small molecule drugs that bind to this surface. If successful, we will have moved from theoretical drug target to lead compound with nanomolar affinity in less than a year.

**P278**

**COMPARISON OF STRATEGIES TO OVERCOME THE HYPERTENSIVE EFFECT OF COMBRETASTATIN-A-4 PHOSPHATE IN A RAT MODEL**

DJ Honess[1*], F Hylands[1], DJ Chaplin[2] and GM Tozer[1]
[1]Tumour Microcirculation Group, Gray Cancer Institute, Mount Vernon Hospital, Northwood, Middx, HA6 2JR, UK and [2]Oxigene Inc., 321 Arsenal St., Watertown, MA 02472, USA.

**Background and Aim.** Combretastatin–A-4 phosphate (CA-4-P) is the lead drug of a group of tubulin depolymerising agents currently in clinical trial as vascular targeting agents. CA-4-P destabilises endothelial cell microtubules and in preclinical models selectively inhibits tumour blood flow (TBF) with subsequent induction of tumour necrosis. However CA-4-P causes transient hypertension prior to the time of maximum inhibition of TBF (Tozer et al, Cancer Res., 1999, **59**, 1626). Clinically, CA-4-P has completed Phase I, with confirmation of TBF reduction by magnetic resonance imaging, and is progressing to Phase II. Patients treated at the maximum tolerated dose also exhibit transient hypertension. If uncontrolled, such hypertensive episodes, could possibly trigger secondary cardiovascular side effects. This study was designed to assess the feasibility of controlling CA-4-P-induced hypertension in rats by means of antihypertensive agents in routine clinical use, the vasodilator sodium nitroprusside (NP) or the β-blocker propranolol (PP).

**Methods and Results.** A tail artery and vein of anaesthetised male BD9 rats were cannulated. Mean arterial blood pressure (MABP) was monitored directly via the arterial cannula, CA-4-P was given ip and NP, PP or saline were given by iv infusion. Mean pre-treatment MABP ± 2SE was 84.1 ± 5.3 mmHg (n = 8). After 30 mg/kg CA-4-P alone MABP rose to 110.3 ± 15.4 mmHg by 20 min (n = 4), remained stable until 80 min, then fell to control values by 120 min. After 10 mg/kg CA-4-P, MABP rose steadily but more slowly to 110.7 ± 11.3 mmHg by 60 min (n = 4), began to fall by 110 min, but remained elevated in 2/4 rats at 120 min. Infusion of 20 μg/kg/min NP reduced MABP to about 60 mmHg and of 40 μg/kg/min PP to about 70

© 2002 Cancer Research UK

mmHg. Saline alone did not alter MABP. CA-4-P at 30 mg/kg was combined with either NP or PP infused at rates up to 20 or 100 µg/kg/min respectively, starting the NP as MABP began to rise, and adjusting infusion rate in response to individual fluctuations. PP elicits its response more slowly, and it proved more effective in maintaining constant MABP to start infusing PP before giving CA-4-P. With the infusion of either NP or PP, MABP was successfully maintained for 120 min within 5 – 10 mmHg of the starting value. When the infusion ended, MABP rose sharply in all NP-treated rats, but in no PP-treated rats. This is most likely due to vasoconstriction on abrupt NP withdrawal or, less probably, to slowing of CA-4-P clearance by a pharmacokinetic interaction between NP and CA-4-P. **Conclusions.** The hypertensive effect of CA-4-P can be successfully abrogated by concomitant infusion with conventional, clinically applicable antihypertensive agents with differing modes of action. This may reduce or eliminate possible cardiovascular side effects of this type of antivascular drug. Prevention of hypertension may also enhance the reduction in specific tumour blood flow, and further animal studies are in progress to investigate this.

*This work was supported by a grant from Oxigene Inc. and Cancer Research UK*

**P279**

**SRC TYROSINE KINASE-DEPENDENT CELLULAR ADHESION CHANGES IN A MODEL OF COLON CANCER METASTASIS**

VG Brunton*, RJ Jones, AW Wyke, E Avizienyte & MC Frame
Cancer Research UK Beatson Laboratories, Garscube Estate, Switchback Road, Bearsden, Glasgow, G61 1BD, UK

One of the key steps leading to the development of metastatic disease is altered cell adhesions, which contribute to cell release from primary sites and establishment of cells at ectopic sites. A common feature of metastatic progression in colon cancer is elevated expression and/or activity of Src tyrosine kinase, although the mechanism of action is unclear. In the Fidler model of human colon cancer metastasis, Src expression and activity is increased in cells that exhibit metastatic potential. In this model, colon cancer cells (KM12C) derived from a primary tumour, metastasise infrequently to the liver when injected into the spleens of mice. However, when cells from these infrequent metastases were subjected to several rounds of injection/metastasis, a highly metastatic variant was derived (KM12L4A). Expression of constitutively active c-Src in the non-metastatic KM12C cells resulted in a dramatic change in morphology. The parental KM12C cells grow as tight clusters with strong cell-cell junctions, while the same cells expressing activated Src have weakened cell-cell contacts and form prominent cellular protrusions which can be reversed by a blocking antibody to $\alpha$v integrin. Integrin engagement in the non-metastatic cells results in the formation of very small, non-protrusive focal complexes, while the metastatic KM12L4A cells and the non-metastatic cells expressing active Src have robust focal adhesions. The formation of these adhesions is delayed in the metastatic cells by expression of kinase defective Src mutants indicating that the Src kinase activity is required. However, under normal growth conditions the KM12L4A cells expressing kinase defective Src mutants also form prominent cellular protrusions similar to those found in the cells expressing constitutively active Src. Thus the kinase activity is not required for the maintenance of these integrin-dependent adhesions and may require the adaptor function of Src mediated by its SH2 and SH3 domains. Using time-lapse video microscopy we found that the protrusions formed in cells expressing active Src were highly motile structures whereas those formed in cells expressing the kinase defective Src protein were static. Thus the kinase activity is required for the dynamic regulation of these integrin-dependent adhesions. Furthermore Src kinase activity is required for the disassembly of cell-cell adhesions as expression of kinase defective Src in the metastatic cells impairs their ability to breakdown cell-cell adhesions in low calcium conditions. Src is found at both cell-matrix and cell-cell adhesions in these colon epithelial cells and it appears that both the kinase activity and adaptor function of Src may play a role in the regulation of these adhesions and that inhibitors of both may be useful therapeutics in the treatment of metastatic disease.

**P280**

**FUNCTIONAL CHARACTERISATION OF DIFFERENTIALLY EXPRESSED GENE TRANSCRIPTS ASSOCIATED WITH COLON CANCER METASTASIS**

E. Foran*, P. McWilliam, D.T. Croke, A.C. Long, Dept. of Biochemistry, Royal College of Surgeons in Ireland, 123 St Stephen's Green, Dublin 2.

Metastasis is the process by which malignant cells leave their primary site and spread to distant locations in the body. Identification of genes that are differentially expressed between primary and secondary tumours is fundamental to the understanding of the metastatic phenotype. Previously we have used two gene expression profiling techniques for this purpose - Suppression Subtraction Hybridization (SSH) and Serial Analysis of Gene Expression (SAGE). These techniques were applied to the isogenic cell lines SW480, a primary tumour, and SW620, a lymph node metastasis from the same patient.

SSH analysis implicated, among other genes, the actin-binding protein L-plastin and several proteasomal subunits in cancer progression. Regulatory and catalytic subunits of the proteasome were down regulated in SW620, the metastatic cell line, with respect to SW480. Following on the initial data obtained using SSH, we tested whether partial inhibition of the proteasome in SW480 had any effect on cell motility and morphology. Basal levels of motility and proliferation were first ascertained, and SW480 was found to be less migratory than SW620. MTS assays showed SW620 to be proliferate at a significantly greater rate than SW480. Treatment of the SW480 cells with the proteasomal inhibitor lactacystin resulted in increased migration of cells, as illustrated by a colloidal gold assay.

Expression of L-plastin at the protein level was significantly greater in the metastatic SW620 cells when compared to SW480. Protein level differences were much greater than RT-PCR analysis of mRNA levels had indicated. The SW480 cell line was stably transfected with L-plastin and functional analysis was carried out. Overexpression of L-plastin increased the proliferation of these cells. Treatment of L-plastin transfected cells with lactacystin resulted in increased expression of L-plastin at the protein level, indicating that L-plastin turnover may be regulated by the proteasome.

These results suggest that differential expression of proteasomal subunits and L-plastin are potentially key regulators of migration and proliferation in these cells and thus markers for tumour progression in a cell line model for colon cancer metastasis.

**P281**

**REFINEMENT OF THE TYLOSIS OESOPHAGEAL CANCER (*TOC*) MINIMAL REGION TO 65KB BY HAPLOTYPE ANALYSIS USING NOVEL MICROSATELITE MARKERS AND SNPS**

Langan JE*[1], Risk JM[1], Field JK[1, 2]
1. Molecular Genetics and Oncology Group, Department of Clinical Dental Sciences, University of Liverpool, Liverpool L69 3GN, UK.
2. Roy Castle International Centre for Lung Cancer Research.

Tylosis is a benign autosomal dominantly inherited disorder of the skin that manifests as focal thickening of the palmar and plantar surfaces. Tylosis is associated with familial oesophageal cancer in two large families in the UK and USA and a smaller German pedigree. The familial cancer association is rare in the general population, but the gene is also thought to be involved in the development of sporadic squamous cell oesophageal cancer. We have previously mapped the Tylosis with Oesophageal Cancer (*TOC*) gene locus to chromosome 17q25 between markers D17S785 and D17S722, spanning a region of 500kb. This region is covered entirely by 4 RPCI-11 BAC clones that are more than 95% sequenced and have been assembled into a contig. The region contains at least 17 candidate genes that were predicted using bioinformatics programs.

In an attempt to further refine the region we have aligned SNPs to the contig and looked for novel microsatellite markers that could be used in haplotype analysis. Over 600 SNPs on the NCBI SNP database aligned with the region (although some proved to be the same SNP) and overlapping sequence data indicated several additional SNPs. Analysis of the sequence data revealed a possible 20 novel microsatellites that ranged from di- to penta-nucleotide repeats. Initial haplotype studies on members of the UK and US pedigrees showed that 9 of the repeats were polymorphic in these families. These novel markers have been submitted to GenBank and have been assigned D numbers by GDB.

© 2002 Cancer Research UK

The 9 novel polymorphic markers were amplified by PCR and characterised by polyacrylamide gel electrophoresis before haplotype analysis in the UK and US families. Two of the new markers, D17S2239 and D17S2244, redefined the TOC minimal region and have narrowed the critical region from ~500kb to just over 65kb. This has not only removed 13 candidate genes from the study but has also eliminated 3 RPCI-11 BAC clones from the minimal contig.

Haplotype analysis of the 25 SNPs on the NCBI database that remain within the TOC minimal region, together with the 20 possible SNPs in this region, is being carried out via a relatively new method called "confronting two pair primers" which will be described. These SNP data will assist in the refinement of the TOC minimal region and may identify disease-specific SNPs in candidate genes.

**P282**

**THE β6 INTEGRIN SUBUNIT C-TERMINAL 11 AMINO ACIDS ARE SUFFICIENT TO PROMOTE INVASION BY SQUAMOUS CELL CARCINOMA (SCC) CELLS**

MR Morgan*, GJ Thomas[1], A Russell[2], PM Speight[1], IR Hart and JF Marshall. Richard Dimbleby Dept of Cancer Research, St Thomas' Hospital, London. [1]Eastman Dental Institute, University of London. [2]Dermatology Dept, Stanford Univ. Medical Centre, Palo Alto, USA

The αvβ6 integrin heterodimer is expressed *de novo* during wound healing and in various epithelial neoplasia. In oral SCC cells, over-expression of αvβ6 *in vitro*, enhances cellular invasion through a ligand-dependent upregulation of secreted pro-MMP-9. We reported previously that the unique C-terminal 11 amino acids of the β6 integrin subunit are critical for αvβ6-dependent MMP-9 upregulation and, as such, are essential for αvβ6-mediated invasion. We investigated whether the C-terminal 11 amino acids of β6 were not only essential for αvβ6-mediated invasion but also sufficient to induce an enhanced invasive phenotype. To address this we used retroviral transduction of cDNAs to generate SCC cell lines over-expressing either wild type β3 or a β3 chimaeric mutant which has its cytoplasmic domain extended by the addition of the C-terminal 11 amino acids of β6. The generated cell lines were V3B3 and V3B3Σ11aa (wild type and chimaeric β3 infectants, respectively), and the null transfectant cell line, C1. Flow cytometry confirmed that the V3B3 and V3B3Σ11aa cells expressed cell surface αvβ3 to the same degree, whereas C1 cells had no αvβ3. Adhesion assays, on vitronectin, showed similar levels of adhesion by the V3B3 and V3B3Σ11aa cell lines (38.60% and 39.45% respectively) which was enhanced relative to C1 null transfectant cells (6.52%). Adhesion of V3B3 and V3B3Σ11aa cells to vitronectin was inhibited by LM609 (anti-αvβ3 blocking antibody) by 30.62% and 26.01%, respectively, but did not affect C1 cell adhesion (6.66%). Interestingly, combined antibody blockade of both αv and β1 reduced V3B3 and V3B3Σ11aa adhesion further (50.29% + 67.7% inhibition respectively). Haptotactic migration assays, towards vitronectin, showed that the V3B3 and V3B3Σ11aa cells attained similar levels of substrate specific haptotaxis (20.29% and 21.79%), whereas C1 cells displayed only basal levels of migration (2.01%). V3B3 and V3B3Σ11aa migration was reduced to basal levels in the presence of LM609 (3.92% and 0%). These data indicated that the wild type and mutant αvβ3 heterodimers, in V3B3 and V3B3Σ11aa cells, are functional as vitronectin receptors. Invasion, through Matrigel, of V3B3Σ11aa cells (131.17%) was significantly higher than that of the V3B3 cell line (100%) (p=0.011). Surprisingly, gelatin zymography demonstrated no significant difference in MMP-9 expression, between V3B3 and V3B3Σ11aa (p=0.164), but the V3B3Σ11aa cells secreted substantial quantities of pro-MMP-2, whereas, the V3B3 cells did not produce a detectable amount. Therefore, the C-terminal 11 amino acids of the β6 integrin subunit, depending on their heterodimer context, are sufficient to enhance SCC cell invasion by promoting either gelatinase A (MMP-9) or B (MMP-2) production.

**P283**

**NOVEL CROSS TALK BETWEEN MEK AND S6K2 IN FGF-2 INDUCED PROLIFERATION OF SCLC CELLS**

Pardo O.E., Arcaro A., Seckl M.J., Lung Cancer Biology Group, Division of Medicine, Hammersmith Campus of Imperial College School of Medicine, London W12 ONN, UK.

Here, we show that fibroblast growth factor-2 (FGF-2) induces proliferation of H510 and H69 small cell lung cancer (SCLC) cells. However, selective activation of the mitogen-activated protein kinase kinase (MEK) pathway was seen in H510, but not H69 cells. Moreover, inhibition of MEK with PD098059 blocked FGF-2-induced proliferation in H510 cells only. Similarly, ribosomal protein S6 kinase 2 (S6K2), a recently identified homologue of S6K1 was activated by FGF-2 in H510, but not H69 cells. This correlated with increased sensitivity of H510 cells to FGF-2 as compared to H69 cells. S6K2 activation was independent of phosphatidylinositol-3 kinase, but was sensitive to inhibition of the MEK pathway. These data suggest that S6K2 is a novel downstream target of MEK. In contrast to S6K2, S6K1 was activated in both SCLC cell lines. Inhibition of the mammalian target of rapamycin with 10 ng/ml rapamycin blocked S6K1 activation and proliferation of both lines. However, even at 100 ng/ml, rapamycin only partially inhibited S6K2. Strikingly, this correlated with inhibition of MEK signalling. Our data indicate that S6K1, and possibly S6K2, are involved in FGF-2-induced SCLC cell growth, a notion supported by the overexpression and higher baseline activity of both isoforms in SCLC lines, as compared to normal human type-II pneumocytes.

**P284**

**INTERLEUKIN-12 POLYMORPHISMS AND GASTRO-OESOPHAGEAL CANCER**

EJ Dickson, SC Cairns, J Eskdale, G Gallagher, RC Stuart.
University Dept of Surgery, Glasgow Royal Infirmary, Glasgow.

**AIM:** The host immunological response to insult depends upon a balance between anti-inflammatory cytokines (e.g.interleukin-10) and pro-inflammatory cytokines such as interleukin-12. A previous study by this group demonstrated that the IL-10R2 allele is over-represented in H. pylori negative gastric cancer.

We therefore decided to test whether there were any associations of IL-12 with gastro-oesophageal cancer.

**METHODS:** DNA was prepared from 135 patients with gastric or oesophageal cancers and 95 normal controls. The samples were genotyped using allele-specific primer polymerase chain reaction, and the results were then analysed with respect to tumour type and H. pylori status.

**RESULTS:** There were no differences in allele frequencies between the cancer and the control groups (80% allele A, 20% allele C). The genotype frequencies were also similar between the two groups (p=0.9) and were not influenced by histological tumour type. However, in the H. pylori-positive group 71.1% of patients were genotype A/A and 26.7% were A/C compared to 51.4% A/A and 42.9% A/C in the H. pylori-negative group (p=0.07).

**CONCLUSIONS:** Our results suggest that there is no significant relationship between this IL-12 marker and histological tumour type. However the trend to significance at this locus implicates a probable role for the IL-12 p40 C allele in H. pylori negative gastro-oesophageal cancer.

**P285**

**PYRIMIDINE METABOLISM AND G1/S TRANSITION IN COLON TUMOURS.**

Collie-Duguid ESR[1*], McKay JA[1], Ladner RD[3], Lynch FJ[4], Ross VG[2], McIntosh RA[1], Murray GI[2], Cassidy J[1].
Departments of Medicine and Therapeutics[1] and Pathology[2], University of Aberdeen, Foresterhill, Aberdeen, Scotland; Department of Molecular Biology, University of Medicine and Dentistry of New Jersey, School of Osteopathic Medicine, Stratford, New Jersey, USA[3]; QualTek Molecular Laboratories Inc., Santa Barbara, CA, USA[4].

Availability of cellular pyrimidine nucleotides is critical for DNA synthesis and the cellular response to perturbations in pyrimidine nucleotide pools is

 © 2002 Cancer Research UK

governed by the status of the cell cycle clock. The relationship between key pyrimidine metabolic enzymes and G1/S transition proteins, and the role of these G1/S pathways in colon cancer prognosis, was investigated. Immunohistochemical analysis of colon tumour cells demonstrated $p21^{WAF1}$ protein expression correlated with TP protein expression (p=0.005). Cytoplasmic dUTPase (p=0.05) or $p27^{KIP1}$ (p=0.031) protein expression was directly correlated with DPD. Cytoplasmic dUTPase (p=0.05), cyclin D1 (p=0.01), p53 (p=0.02) or $p21^{WAF1}$ (p=0.001) protein expression was associated with nuclear dUTPase protein expression.

Nuclear dUTPase protein was an independent predictor of survival in Dukes' C colon cancer, where patients expressing this isoform had a poorer survival than those negative for nuclear dUTPase (Odds ratio 4.1; 95 % CI 1.3-12.3; p=0.01). Multivariate analysis of combined enzyme expression demonstrated Dukes' C colon cancer patients with positive expression of both nuclear dUTPase and TP protein in their tumour cells have a highly increased risk of death compared to patients with positive expression of only one or none of these enzymes (Odds ratio=13.2, 95% CI=3.2-54.3, p<0.001). Patients (Dukes' A, B, C and D) with colon tumour cells positively expressing nuclear dUTPase in combination with either p53 (Odds ratio 4.7, 95% CI 1.5-14.4, p=0.007) or p21 (Odds ratio 2.9, 95% CI 1.2-6.8, p=0.017) have poorer survival than those expressing one or none of these proteins.

© 2002 Cancer Research UK

